# Eight years after an international workshop on myotonic dystrophy patient registries: case study of a global collaboration for a rare disease

**DOI:** 10.1186/s13023-018-0889-0

**Published:** 2018-09-05

**Authors:** Libby Wood, Guillaume Bassez, Corinne Bleyenheuft, Craig Campbell, Louise Cossette, Aura Cecilia Jimenez-Moreno, Yi Dai, Hugh Dawkins, Jorge Alberto Diaz Manera, Celine Dogan, Rasha el Sherif, Barbara Fossati, Caroline Graham, James Hilbert, Kristinia Kastreva, En Kimura, Lawrence Korngut, Anna Kostera-Pruszczyk, Christopher Lindberg, Bjorn Lindvall, Elizabeth Luebbe, Anna Lusakowska, Radim Mazanec, Giovani Meola, Liannna Orlando, Masanori P. Takahashi, Stojan Peric, Jack Puymirat, Vidosava Rakocevic-Stojanovic, Miriam Rodrigues, Richard Roxburgh, Benedikt Schoser, Sonia Segovia, Andriy Shatillo, Simone Thiele, Ivailo Tournev, Baziel van Engelen, Stanislav Vohanka, Hanns Lochmüller

**Affiliations:** 1 Institute of Genetic Medicine, Newcastle University, Newcastle upon Tyne, UK; 2 Centre de référence des maladies neuromusculaires, Hôpital Henri Mondor, Paris, France; 3 Scientific Institute of Public Health, Brussels, Belgium; 4 Western University, London, Canada; 5 Centre de recherche du CHU de Québec, Université Laval, Quebec, Canada; 6 Department of Neurology, Peking Union Medical College Hospital, Chinese Academy of Medical Sciences, Beijing, China; 7 Office of Population Health Genomics, Perth, Western Australia; 8 Neuromuscular disorders Unit, Hospital de la Santa Creu I Sant Pau, Barcelona, Spain; 9 Neuromuscular & Neuro-genetics Unit, Air Hospital, Cairo, Egypt; 10 U.O. Neurology and Stroke Unit, IRCCS Policlinico San Donato, San Donato Milanese, Milan, Italy; 11 Department of Neurology, University of Rochester Medical Center, Rochester, NY USA; 12 Department of Neurology, Alexandrovska University Hospital, Medical University, Sofia, Bulgaria; 13 Department of Promoting Clinical Trial and Translational Medicine, National Center for Neurology and Psychiatry, Translational Medical Center, Kodaira, Japan; 14 University of Calgary, Calgary, Canada; 15 Department of Neurology, Medical University of Warsaw, Warszawa, Poland; 16 University Hospital Örebro, Örebro, Sweden; 17 University Hospital Prague- Motol and Charles University Prague, Prague, Czech Republic; 18 Muscular Dystrophy Association, Chicago, USA; 19 Department of Functional Diagnostic Science, Osaka University Graduate School of Medicine, Suita, Japan; 20 Neurology Clinic, School of Medicine, University of Belgrade, Belgrade, Serbia; 21 Neurology, Auckland City Hospital, Private Bag 92024, Auckland, 1142 New Zealand; 22 Friedrich-Baur-Institute, Department of Neurology, Klinikum München, Munich, Germany; 23 Centro de Investigación Biomédica en Red en Enfermedades Raras (CIBERER), Valencia, Spain; 24 Institute of Neurology, Psychiatry and Narcology, Academy of medical science of Ukraine, Kharkiv, Ukraine; 25 Radboud University Nijmegen Medical Centre, Nijmegen, Netherlands; 26 University Hospital and Masaryk University Brno, Brno, Czech Republic; 27 Department of Neuropediatrics and Muscle Disorders, Medical Center, Faculty of Medicine, University of Freiburg, Freiburg, Germany; 28 Centro Nacional de Análisis Genómico (CNAG-CRG), Center for Genomic Regulation, Barcelona Institute of Science and Technology (BIST), Barcelona, Spain

**Keywords:** Myotonic dystrophy, Registries, Clinical trials, Trial readiness

## Abstract

**Background:**

Myotonic Dystrophy is the most common form of muscular dystrophy in adults, affecting an estimated 10 per 100,000 people. It is a multisystemic disorder affecting multiple generations with increasing severity. There are currently no licenced therapies to reverse, slow down or cure its symptoms. In 2009 TREAT-NMD (a global alliance with the mission of improving trial readiness for neuromuscular diseases) and the Marigold Foundation held a workshop of key opinion leaders to agree a minimal dataset for patient registries in myotonic dystrophy. Eight years after this workshop, we surveyed 22 registries collecting information on myotonic dystrophy patients to assess the proliferation and utility the dataset agreed in 2009. These registries represent over 10,000 myotonic dystrophy patients worldwide (Europe, North America, Asia and Oceania).

**Results:**

The registries use a variety of data collection methods (e.g. online patient surveys or clinician led) and have a variety of budgets (from being run by volunteers to annual budgets over €200,000). All registries collect at least some of the originally agreed data items, and a number of additional items have been suggested in particular items on cognitive impact.

**Conclusions:**

The community should consider how to maximise this collective resource in future therapeutic programmes.

**Electronic supplementary material:**

The online version of this article (10.1186/s13023-018-0889-0) contains supplementary material, which is available to authorized users.

## Background

Patient Registries have the different objectives that include: [1] improve the understanding of the prevalence, natural history, pathogenesis, and treatment options of diseases in recognition by a systematic collection of clinical and demographic data; [2] set up an infrastructure that allows collaboration between patients, health and care providers, academia (research) and industry; and, [3] obtain a picture of the targeted cohort’s real-life disease burden, standards of care and patient’s preferences towards treatment [1–3].

In rare diseases such as Myotonic Dystrophy (DM), Disease-specific Patient Registries have been recognised as indispensable tools to translate clinical and research knowledge into therapeutic solutions [1, 4–6]. DM is a genetically caused neuromuscular condition estimated to affect 10 per 100,000 people in European populations [7, 8]. It is found in two forms: myotonic dystrophy type 1 (DM1) and the less frequent myotonic dystrophy type two (DM2). DM1 is one of the most variable human diseases with a complex multisystemic presentation. The condition is characterised by progressive muscle wasting and myotonia; however, it also has significant impact on cognition cardiac, visual, endocrine, gastrointestinal, and pulmonary systems. These manifestations have a significant effect impact on social participation and quality of life [9–13] . There are currently limited therapies for certain symptoms, and none to slow down, reverse or cure the disease. As with all rare diseases, the need to coordinate resources and knowledge is essential in order to move towards larger and more successful clinical trials and ultimately better management.

The International Rare Diseases Research Consortium (IRDiRC) advocates for a global collaboration between different disease specific registries collecting a minimum set of standardised and consistent data that will boost research at all levels [14]. In 2009, a group of key opinion leaders agreed upon a minimal core dataset for DM patient registries. This dataset was agreed at a TREAT-NMD/Marigold workshop held in Naarden, Netherlands and has since been referred to as the “Naarden” dataset [15, 16]. Since then a unified global registry for DM, using a federated or centralised model has not been fully established. However, an increasing number of registries are collecting what was agreed as essential data on myotonic dystrophy. Here we present a summary of the global experience of designing and setting up these registries. We aim to describe the current landscape of DM registries and to assess how widely used the Naarden dataset is and its perceived usefulness or shortcomings.

## Methods

**Fig. 1:**
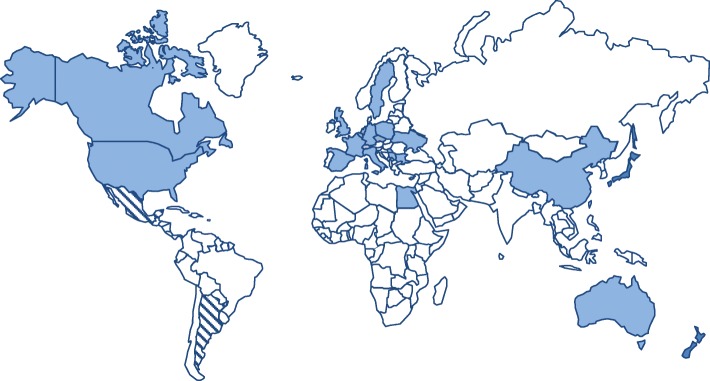
Location of registries collecting data on myotonic dystrophy. The countries shaded with stripes are in the set up phase of registries

Through the platforms of TREAT-NMD (global alliance with the mission of improving trial readiness in neuromuscular diseases) and the Marigold Foundation (a research foundation DM-specific who commissioned this report), 25 registries were identified worldwide and invited to participate (Fig. 1). All registries were invited to complete an online survey asking about the design, set up, and utility of the registry. The original data was collection period was between September and December 2015, with updates provided in April 2017. This survey can be found in the Additional file 1.

## Results

Of these 25 registries, 21 were currently active, two had recently launched and two were still in set up phase. Twenty-two registries responded to this request, and it is this information that is described here.

Collectively these registries contain information on over 10,000 DM patients worldwide (Table 1). The longest standing registry is the “National Registry for Myotonic Dystrophy and Facioscapulohumeral Muscular Dystrophy Patients and Family Members” based at the University of Rochester, USA which started collecting data in 2000 [17]. A further six registries/databases were established before the publication of the original Naarden dataset (CRAMP, Genemu, BNMDR and DM-SCOPE and the registries in Serbia and Bulgaria). In the years since this workshop, an additional 15 registries collecting data on DM patients have been launched.

**Table 1:** Numbers of patients registered across countries as provided in April 2017

Name of Registry	Country	Year established	DM1 > 18	DM1 < 18	DM2 > 18	DM2 < 18
National Registry for Myotonic Dystrophy and Facioscapulohumeral Muscular Dystrophy Patients and Family Members	USA	2000	1177	67	214	0
CRAMPS	Netherlands	2001	452	0	30	0
Genemu**	Quebec, Canada	2005	N/A	N/A	N/A	N/A
Belgian Neuromuscular Disease Registry	Belgium	2008	493	34	16	2
DM-SCOPE	France	2008	2203	255	107	0
Bulgarian Myotonic Dystrophy Registry	Bulgaria	2009	76	2	6	0
Akhenaten, Serbian Registry for Myotonic Dystrophies	Serbia	2009	335	0	86	0
Polish Registry of Neuromuscular Patients	Poland	2010	246	7	125	0
ReaDY Myotonic Disorders	Czech Republic	2011	184	5	286	2
Canadian Neuromuscular Disease Registry	Canada	2011	188	21	38	0
New Zealand Neuromuscular Disease Registry	New Zealand	2011	156	11	11	0
Myotonic Dystrophy Patient Registry for Germany and Switzerland	Germany and Switzerland	2012	243	16	246	3
UK Myotonic Dystrophy Patient Registry	UK	2012	429	45	16	0
China DM Registry	China	2012	61	8	0	0
Spanish Registry of neuromuscular diseases	Spain	2012	265	6	7	0
Egyptian neuromuscular registry	Egypt	2013	4	6	5	3
The Italian registry for Myotonic Dystrophy	Italy	2013	491	16	31	0
Myotonic Dystrophy Family Registry*	USA	2013	1051*		250*	
NMiS	Sweden	2013	194	17	0	0
Registry of Muscular Dystrophy REMUDY	Japan	2014	554	45	1	0
Ukrainian registry of muscular dystrophies	Ukraine	Not provided	3	1	0	0
Australian Myotonic Dystrophy Registry***	Australia	Data collection has not yet begun	0	0	0	0
Totals			9156		1485	
Total registered globally			10,641			

*Estimates based on literature review. **Recent numbers not available. ***Data collection not yet begun

In many cases registries are collecting information on both DM1 and DM2. Across the registries surveyed, the majority of records (86%) related to DM1 patients. However, there are some notable exceptions to this only 40% of patients in the German registry have DM1, and 51% in ReaDY (Czech Republic). A larger proportion of DM2 patients are also present in Akhenaten (Serbia) and REMUDY (Japan) where 33% and 20% of registered patients have DM2.

### Mandatory and highly encouraged items

The mandatory and highly encouraged items from the “Naarden” dataset collected by each registry are summarised in Table 2. All registries collected at least 50% of the mandatory items as listed in the original core dataset with 13 (59%) collecting all of these mandatory items. Eight (36%) registries collected all of the mandatory and highly encouraged items. Two of the registries set up before the publication of the Naarden dataset (Genemu and the Bulgarian registry) already collected all of the mandatory data items. Ten of the 15 registries launched after the publication of the Naarden dataset collect all of these items.

**Table 2:**
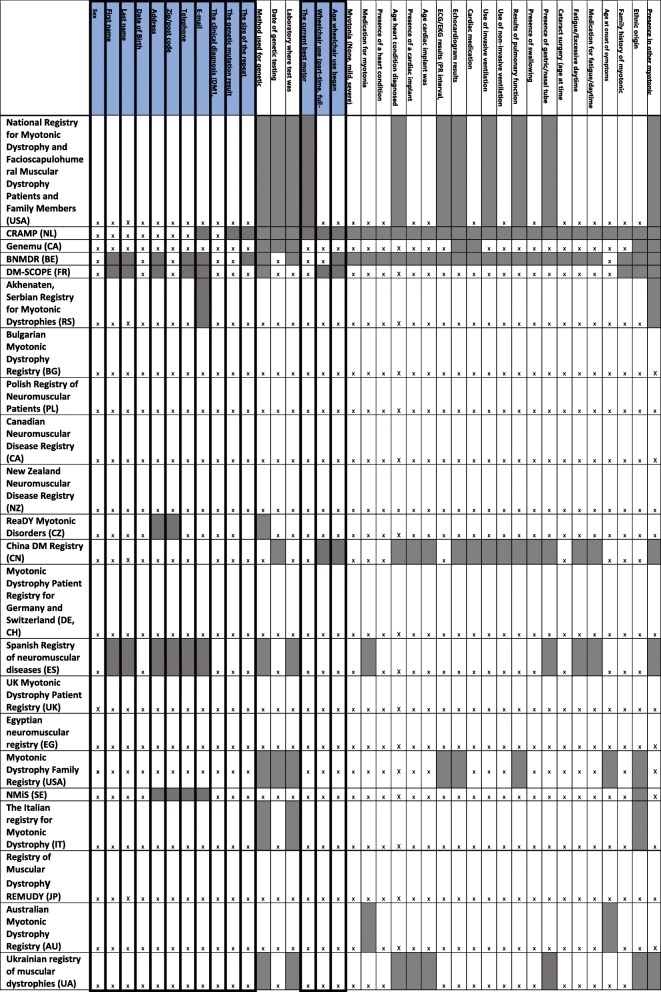
Proliferation of the core “Naarden” dataset for myotonic dystrophy registries

“X” indicates the item is collected. The highlighted data items those listed as mandatory in the Naarden dataset. The shading indicates that these items are not collected by the registry

Only 12 (55%) of the 22 registries surveyed asked participants whether they were signed up to another registry to determine if there are duplicate registrations. Registry owners were asked which items they would consider removing from the original core dataset, two registry curators answered that ethnic origin was not essential, two suggested the removal of ECG and echocardiogram results and two suggested that the date and method used for genetic testing should be removed.

Along with the core dataset, many registries collect additional information about myotonic dystrophy patients; some registries use additional validated questionnaires relating to pain (McGill, BI, NSPI), fatigue (ESS, FDSS) and quality of life (SF36, InQoL, EQ. 5D and ActivLim). In addition, some registries collect data regarding the gastrointestinal and central nervous systems and anthropometric measurements. Several registries also include detailed socioeconomic data. However, there was no standardisation or commonality among these items.

### Purpose and utility of the registries

**Fig. 2:**
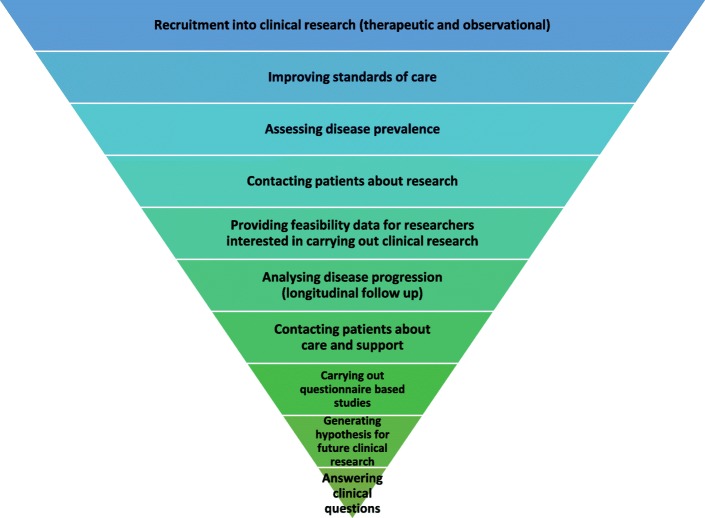
The registry purpose ranked from most to least importance by registry owners

The registries and databases contacted were asked to rank 10 possible purposes of registries in order of importance. The most common purpose was for recruitment into clinical research both for therapeutic and observational studies. Providing feasibility data to researchers and improving standards of care were also among top priorities. Assessing disease prevalence and analysis of disease progression were less often the main purpose (Fig. 2).

Thirteen (59%) of the 22 registries reported having supported recruitment into clinical research, 11 had provided feasibility data for use in planning of a trial and 13 reported entering recruitment of patients to questionnaire based studies. Eleven (50%) of the registries had contributed to publications in scientific, peer-reviewed journals. This includes publications resulting from collaborations between registries, such as a recent paper from the UK, Germany and Dutch groups [18].

#### Registry characteristics

Characteristics of these registries composition are summarised in Table 3.

**Table 3:** Configuration and set up of the 22 registries actively collecting data on myotonic dystrophy patients

	Coverage of Registry	Scope of Registry	Funding Mechanism	Annual running costs	Data collection tool	Who enters the data
National Registry for Myotonic Dystrophy and Facioscapulohumeral Muscular Dystrophy Patients and Family Members (USA)	USA	DM1,DM2,FSHD	Grant or Project funding	Unknown	Paper Electronic database (Access, SAS)	Healthcare professional (nurse, clerk, research assistant) Patients Clinical specialists
CRAMPS	Netherlands	All neuromuscular diseases	Healthcare System	Unknown	Electronic database	Clinical specialists
Genemu	Quebec	DM1, DM2	Grant or Project funding	€23,000	N/A	Healthcare professional (nurse, clerk, research assistant)
BNMDR	Belgium	All Neuromuscular diseases	Healthcare system	€ 145,000	Electronic database (healthdata.be) Paper	Healthcare professional (nurse, clerk, research assistant) Clinical Specialists
DM-SCOPE	France	DM1, DM2	Charity	€140,000	N/A	Healthcare professional (nurse, clerk, research assistant) Clinical specialists Geneticist (diagnostic lab)
Akhenaten, Serbian Registry for Myotonic Dystrophies	Serbia	DM1, DM2	Grant or Project funding	Unknown	Electronic database	Clinical specialists
Bulgarian Myotonic Dystrophy Registry	Bulgaria	DM1, DM2	N/A	€ 0	Electronic database (Excel)	Clinical specialists
Polish Registry of Neuromuscular Patients	Poland	DM1, DM2, SMA, DMD/BMD	Project Grant	€0	Paper Electronic database	Clinical specialist
Canadian Neuromuscular Disease Registry	Canada	All neuromuscular diseases	Industry, Charity, Grant or project funding	€ 200,000	Electronic database (custom solution)	Healthcare professional (nurse, clerk, research assistant)
New Zealand Neuromuscular Disease Registry	New Zealand	All neuromuscular diseases	Charity	€ 30,000	Electronic database (RDRF)	Healthcare professional (nurse, clerk, research assistant) Patients
ReaDY Myotonic Disorders	Czech Republic	All myotonic disorders	Charity	€10,000	Electronic database (Oracle)	Clinical specialists
China DM Registry	China	All Neuromuscular diseases	Healthcare system	€7500		
Myotonic Dystrophy Patient Registry for Germany and Switzerland	Germany and Switzerland	DM1, DM2	Unknown	Unknown	Electronic database	Clinical specialists General practitioners Patients
Spanish Registry of neuromuscular diseases	Spain	All Neuromuscular diseases	Grant or project funding	€ 50,000	Electronic database (SQL)	Clinical specialists
UK Myotonic Dystrophy Patient Registry	UK	DM1, DM2	Charity Grant of project funding	€ 15,000	Electronic database (custom solution)	Healthcare professional (nurse, clerk, research assistant) Patients Clinical specialists
Egyptian neuromuscular registry	International	All Neuromuscular diseases	Healthcare system	€200	Paper	Clinical specialists
Myotonic Dystrophy Family Registry	International	DM1, DM2	unknown	unknown	Electronic database (patient crossroads)	Patients Family members
NMiS	Sweden	All inherited myopathies	Government funding	€ 10,000	Electronic database	Clinical specialists
The Italian registry for Myotonic Dystrophy	Italy	DM1, DM2	Grant or project funding.	€ 30,000	Electronic database	Patients Clinical specialists
Registry of Muscular Dystrophy REMUDY	Japan	All Neuromuscular diseases	Grant of project funding	€420,000	Paper Electronic database (custom solution)	Patients Clinical specialists
Australian Myotonic Dystrophy Registry	Australia	DM1, DM2	Government, Hospital and Clinical groups.	€ 12,954	Electronic database	Clinical specialists Healthcare professional (nurse, clerk, research assistant) Geneticist (diagnostic lab)
Ukrainian registry of muscular dystrophies	Ukraine	All neuromuscular diseases	Charity	Unknown	Paper Electronic database (excel)	Clinical specialists General practitioners Patients

The majority of the registries surveyed have national coverage; two (Myotonic Dystrophy Family Registry – USA and the Egyptian neuromuscular registry) accept international registrations. Ten (46%) of the registries surveyed are myotonic dystrophy specific while the others are collecting information on all neuromuscular diseases or a selection of other conditions (i.e. USA and Poland).

### Resources and technical solutions

Our survey indicated that there is no coordinated or central funding for DM patient registries. Funding is typically obtained on a national level and is often a combination of patient organisations or other charitable funding alongside grant or project funding. One registry receives some funding from industry (Canadian Neuromuscular Disease Registry) in addition to other sources, while six (Australia, BNMDR, China, CRAMP, Egypt, and Italy) receive funds from the healthcare system or government.

The financial resources required to set up and maintain the registries were not provided in all cases. From the information available the best-resourced registry is the Japanese registry of muscular dystrophy (REMUDY) with set up costs of €420,000; this is followed but the Canadian Neuromuscular Disease Registry (CNDR with set up costs of €240,000 ($337,000 CAD) and annual running costs of €250,000 ($350,000 CAD). Notably both registries collect information from a range of neuromuscular diseases and these resources are not dedicated to myotonic dystrophy. At the other end of the scale, there is the Bulgarian Myotonic Dystrophy Registry, which has received no funding and is managed through free donation of the team’s time. A number of other registries have received funding to set up the registry but this has not been sustained and they are now running on limited or no funds. Not considering these outliers, the mean set up cost was €53,100 (range €1000 – € 145,000) and the annual running costs €200 to €145,000 with a mean of €49,711.55.

This diversity in funding is reflected in the data capture methods implemented with no two registries using the same IT solutions, and some using hard copy data collection methods. The electronic solutions vary from excel spreadsheets to complex custom software systems.

### Data entry

Data is most often updated annually, with most registries striving for annual updates though some stating this is not always possible due to resource limitations. A number of registries (Ukraine and Egypt) update details on a six-monthly basis.

All but one of the surveyed registries involves data entry by a clinician or another healthcare professional. Only the Myotonic Dystrophy Family Registry (USA) is entirely patient reported. However, an element of patient reported data is included in seven additional registries (UK, Italy, and USA National Registry at the University of Rochester, Japan, Germany, New Zealand and Ukraine). Several registries (DM-Scope and Australian DM registry) also allow data entry from the diagnostic laboratory.

#### Alternative cohorts

Throughout this process it has been acknowledged that there are many additional cohorts with valuable data on myotonic dystrophy patients; this includes data being collected in natural history studies, clinical trials and by patient organisations and mailing lists. Two of the most significant of these so-called “alternative” cohorts, are held by the myotonic dystrophy support group (MDSG) in the UK and the Muscular Dystrophy Association (MDA) in the USA. MDSG collects contact details of families in the UK, this is allows them to provide a quarterly newsletter and act as a central point for of information and support. There are currently more than 2500 families registered with MDSG, this database does not contain clinical or symptomatic details. MDA holds information on over 10,500 people with a diagnosis of myotonic dystrophy (DM1 and DM2), the majority of whom are adults. Developed initially as contact databases, these therefore does not collect the Naarden dataset, however they capture a number of socio economic items (insurance coverage, employment status, income range and marital status) relevant for analysis. These cohorts share challenges with the more traditional registries in terms of resources to ensure data quality and governance.

## Discussion

Patient Registries are a key concern for all those involved in the rare diseases field, however only about a fifth of rare diseases have registries [19]. DM is one of these rare diseases with a clear interest and investment in registries. These registries represent over 10,000 DM patients which represents a promising resource to support trial readiness aims such as feasibility and recruitment and the importance of cross international boundaries in this topic. This overview presents a summary of the global infrastructure currently available for DM and highlights points of agreement between different registries and caveats that can be improved to allow efficient future collaborations.

### Utility and proliferation of the “Naarden Dataset”

Despite the variability seen across registries, in set-up, purpose and execution many of the items of the original dataset remain relatively consistent across resources. The impact can be seen in the number of new registries (66%) having adopted all of the mandatory items since 2009, compared to the 28% of registries that collected these items prior to the Naarden workshop. The fact that there was no agreement on items to be removed or added suggests that the “Naarden” dataset may still be an appropriate minimal dataset for myotonic dystrophy patient registries. It is important to note that there is significant number of registries not yet collecting all items of the core dataset. This may reflect the variety of resources available to the registries. However, it may also indicate a need to increase awareness of the dataset and to better highlight the added value to of collecting this data in a harmonised way. Yet, with many registries collecting items beyond the previously agreed dataset an expansion of the dataset could be considered, in particular the addition of anthropometric measurements and items looking at cognitive impairment. Furthermore, the higher frequency of DM2 patients in some areas of central Europe may also call for an adapted dataset accounting for differences in phenotypic presentation. With more patients entering clinical trials over the coming years, this should be captured in additional questions.

The growing number of registries collecting additional patient reported outcomes also calls for further consideration. A significant amount of work has already been done towards the selection and harmonisation of disease-specific reported outcomes by the Outcome Measures for Myotonic Dystrophy initiative (OMMYD) [20, 21]. The registry community should consider adopting these guidelines to ensure only tools validated in these disease areas are included. We also recognise the importance of including patient representation when refining the outcomes most appropriate to include in registries.

The numbers of research studies and publications derived from the registries demonstrate the utility of the registries. This is best demonstrated in the multicentre clinical trial setting of OPTIMISTIC [22], in which the registries from the UK, France, Germany and the Netherlands were successfully used for feasibility and recruitment. This experience together with tools already developed by TREAT-NMD mean that the myotonic dystrophy registry community is well placed to help facilitate and accelerate clinical research.

### Future harmonisation and interoperability

There is a clear need for a more integrated approach to DM registries; however, the building blocks for increased collaboration are in place. The registries identified, share common data items and have the same fundamental purpose. All of the registries we contacted were open to the idea of increased collaboration. However, the diversity in registry collection methods may present a bottleneck when looking to future interpretability. As part of a recent collaboration between three of these registries surveying about falls and related injuries, minor differences between collected demographic data limited the full interpretability of the results from the questionnaire [18]. Within the wider rare disease registry community there is a call for more standardised collection of data using ontologies and for data to be meet the FAIR (Findable, accessible, interoperable and reusable) data principles [23, 24]. Interoperability of these datasets could also be addressed through a common identifier, or privacy protection record linkage (PPRL). The myotonic dystrophy community is well placed to be part of these developments.

Some investment would be required in order for the existing registries to meet these standards; however, a significant amount of work in this area is being carried out in some larger projects such as RD-Connect [25], who are looking to demonstrate how different datasets including registries can be linked. Beyond connecting registries with each other, increased value could come from further linking registry data to biobanks, natural history and omics data for example. Furthermore, these larger initiatives should be utilised to increase the visibility of these registries, for example, currently a search of the Orphanet catalogue of rare disease registries listed only 9 DM registries while the RD-Connect Registry and Biobank Finder system returns 13 results.

It would be unrealistic to expect that a single individual or group will fully lead the establishment of a global and unique rare-disease registry. Patient registries are dependent on the availability of resources (human and technology), standards of care and research and specific regulatory bodies or criteria [1, 4, 26]. However, this report can be considered a rare disease case-study about the success of collaborating at a global level and the importance of establishing a set of data considered essential for a disease-specific patient registry.

## Conclusion

In conclusion, there is a desire within the myotonic dystrophy community to better unite the registry landscape and increase interoperability. Consideration will have to be given to the resources available for this to ensure sustainability. In addition, it is important to see how these registries fit in the bigger picture of the rare disease community. The registries contacted are eager to increase and continue this collaboration, a follow-up workshop might be required to work towards meeting the goals of standardisation and interpretability. Such a workshop could also be used to better address the impact of the data set in the current trial landscape. The networks provided by TREAT-NMD, RD-Connect, and the International Rare Diseases Research Consortium (IRDiRC) represent access to the necessary tools and stakeholders to ensure the greatest impact of these valuable resources. The utility could be further demonstrated through analysis of the data within all of these registries. Analysis on the 10,000 patients would provide an insight into this condition on a scale not previously seen.

### Highlights

- Disease-specific Patient Registries are indispensable platforms to succeed with therapeutic solutions.
- Over 10,000 myotonic dystrophy patients captured in registries worldwide.
- Twenty-two registries collect a comparable dataset on myotonic dystrophy patients.
- There is still a huge variety in data collection and funding mechanisms among registries.
- Significant clinical research has been supported by these myotonic dystrophy registries.

## Additional file

Additional file 1: Survey to capture details about the registry, database or mailing list. (PDF 270 kb)

## Abbreviations

BNMDR: Belgian Neuromuscular Disease Registry
CNDR: Canadian Neuromuscular Disease Registry
CRAMP: Computer Registry of All Myopathies and Polyneuropathies
DM: myotonic dystrophy
DM1: Myotonic dystrophy type 1
DM2: Myotonic dystrophy type 2
ECG: Electrocardiogram
ESS: Epworth Sleepiness Scale
FAIR: Findable Accessible Interoperable and Reusable
FDSS: Fatigue and Daytime Sleepiness Scale
InQoL: Individualised Neuromuscular Quality of Life
IRDiRC: International Rare Diseases Research Consortium
MDA: Muscular Dystrophy Association
MDSG: Myotonic Dystrophy Support Group
NMiS: Neuromuscular Diseases in Sweden
PPRL: Personal protection record linkage
ReaDY: Registry of Muscular Dystrophy, Czech Republic
REMUDY: Registry of Muscular Dystrophy, Japan
TREAT-NMD: Translational Research in Europe Assessment and Treatment of Neuromuscular Diseases
