# Correction to: Eight years after an international workshop on myotonic dystrophy patient registries: case study of a global collaboration for a rare disease

**DOI:** 10.1186/s13023-019-1157-7

**Published:** 2019-08-15

**Authors:** Libby Wood, Guillaume Bassez, Corinne Bleyenheuft, Craig Campbell, Louise Cossette, Aura Cecilia Jimenez-Moreno, Yi Dai, Hugh Dawkins, Jordi Díaz-Manera, Celine Dogan, Rasha el Sherif, Barbara Fossati, Caroline Graham, James Hilbert, Kristinia Kastreva, En Kimura, Lawrence Korngut, Anna Kostera-Pruszczyk, Christopher Lindberg, Bjorn Lindvall, Elizabeth Luebbe, Anna Lusakowska, Radim Mazanec, Giovani Meola, Liannna Orlando, Masanori P. Takahashi, Stojan Peric, Jack Puymirat, Vidosava Rakocevic-Stojanovic, Miriam Rodrigues, Richard Roxburgh, Benedikt Schoser, Sonia Segovia, Andriy Shatillo, Simone Thiele, Ivailo Tournev, Baziel van Engelen, Stanislav Vohanka, Hanns Lochmüller

**Affiliations:** 1 Institute of Genetic Medicine, Newcastle University, Newcastle upon Tyne, UK; 2 Centre de Référence des Maladies Neuromusculaires, Hôpital Henri Mondor, Paris, France; 3 Scientific Institute of Public Health, Brussels, Belgium; 4 Western University, London, Canada; 5 Centre de recherche du CHU de Québec, Université Laval, Quebec, Canada; 6 Department of Neurology, Peking Union Medical College Hospital, Chinese Academy of Medical Sciences, Beijing, China; 7 Office of Population Health Genomics, Perth, Western Australia; 8 Neuromuscular disorders Unit, Hospital de la Santa Creu I Sant Pau, Barcelona, Spain; 9 Neuromuscular & Neuro-genetics Unit, Air Hospital, Cairo, Egypt; 10 U.O. Neurology and Stroke Unit, IRCCS Policlinico San Donato, San Donato Milanese, Milan, Italy; 11 Department of Neurology, University of Rochester Medical Center, Rochester, NY USA; 12 Department of Neurology, Alexandrovska University Hospital, Medical University, Sofia, Bulgaria; 13 Department of Promoting Clinical Trial and Translational Medicine, National Center for Neurology and Psychiatry, Translational Medical Center, Kodaira, Japan; 14 University of Calgary, Calgary, Canada; 15 Department of Neurology, Medical University of Warsaw, Warszawa, Poland; 16 University Hospital Örebro, Örebro, Sweden; 17 University Hospital Prague- Motol and Charles University Prague, Prague, Czech Republic; 18 Muscular Dystrophy Association, Chicago, USA; 19 Department of Functional Diagnostic Science, Osaka University Graduate School of Medicine, Suita, Japan; 20 Neurology Clinic, School of Medicine, University of Belgrade, Belgrade, Serbia; 21 Neurology, Auckland City Hospital, Private Bag 92024, Auckland, 1142 New Zealand; 22 Department of Neurology, Friedrich-Baur-Institute, Klinikum München, Munich, Germany; 23 Centro de Investigación Biomédica en Red en Enfermedades Raras (CIBERER), Valencia, Spain; 24 Psychiatry and Narcology, Academy of medical science of Ukraine, Institute of Neurology, Kharkiv, Ukraine; 25 Radboud University Nijmegen Medical Centre, Nijmegen, Netherlands; 26 University Hospital and Masaryk University Brno, Brno, Czech Republic; 27 Department of Neuropediatrics and Muscle Disorders, Medical Center, Faculty of Medicine, University of Freiburg, Freiburg, Germany; 28 Centro Nacional de Análisis Genómico (CNAG-CRG), Center for Genomic Regulation, Barcelona Institute of Science and Technology (BIST), Barcelona, Spain

**Correction to: Orphanet J Rare Dis (2018) 13:155.**

**https://doi.org/10.1186/s13023-018-0889-0**

The original version of this article [1] unfortunately included an error to an author’s name. Author Jordi Díaz-Manera was erroneously presented as Jorge Alberto Diaz Manera. The correct author name has been included in the author list of this Correction article.

For citation purposes the author’s given name is Jordi and family name Díaz-Manera. Therefore, the correct citation of the author’s details is: Díaz-Manera J.
