# Supplementary material for: Eight years after an international workshop on myotonic dystrophy patient registries: case study of a global collaboration for a rare disease
Source: Orphanet J Rare Dis. 2018 Sep 5;13:155. doi: 10.1186/s13023-018-0889-0 (PMC6126043; doi:10.1186/s13023-018-0889-0)
Supplement: Supplementary file 1 — Survey to capture details about the registry, database or mailing list. (PDF 270 kb) [file 13023_2018_889_MOESM1_ESM.pdf]

1. About your registry, database or mailing list.

* 1. Name of registry, database or mailing list

* 2. Name of organisation that is home to the registry, database or mailing list

* 3. Please provide the contact details of the primary contact:

Name

Institution

Country

Email Address

Phone Number

4. Please provide the contact details for the secondary contact:

Name

Institution

Country

Email Address

Phone Number

5. Website:

2. About your registry, database, cohort or mailing list.

6. Please specify if you are completing this survey as the manager/owner of a registry, database or mailing list. (If your organisation is host to multiple then please complete this questionnaire for each instance)

- ○ A registry
- ○ A database
- ○ A mailing list
- ○ Other (please specify)

[ ]

7. When did data collection begin?

	DD		MM		YYYY
Date:	[ ]	/	[ ]	/	[ ]

* 8. What is the coverage of the registry, database or mailing list?

- ○ International
- ○ National (Please specify country below)
- ○ Regional (Please specify region below)

Please specify country or region

[ ]

* 9. Range of diseases covered

- ○ Myotonic dystrophy type 1 only
- ○ Myotonic dystrophy type 2 only
- ○ Myotonic dystrophy type 1 and 2
- ○ All Neuromusclar diseases
- ○ All rare diseases
- ○ Other (please specify)

[ ]

10. How is the registry, database or mailing list funded? (please select all options that apply)

- [ ] Industry
- [ ] Patient organisation (advocacy or support group)
- [ ] Charitable foundation
- [ ] Grant or project funding
- [ ] Healthcare system- hospital or clinical operating funds
- [ ] Other government funding
- [ ] Other (please specify)

11. Please estimate the initial set-up costs of the registry, where possible please provide these costs in Euro (€).

12. Please estimate the ongoing costs, per annum for the maintenance of the registry. Where possible please provide these costs in Euro (€)

3.

* 13. Who enters data into the registry, database or mailing list? (please tick all that applies)

- [ ] Patients
- [ ] Clinicians (specialists)
- [ ] Clinicians (family doctor or general practitioner)
- [ ] Other healthcare professionals (nurse, clerk, research assistant)
- [ ] Geneticist (from diagnostic laboratory)
- [ ] Other (please specify)

14. How often is data captured?

- ( ) Every six months
- ( ) Annually
- ( ) Data is only provided once.
- ( ) Ad Hoc (please provide details below)

Other (please specify)

15. How is data stored?

- [ ] Paper copy- within clinical notes
- [ ] Paper copy- separate to clinical notes
- [ ] Electronically within a database
- [ ] Electronically in an online cloud based service
- [ ] Electronically on a laptop or desktop computer
- [ ] Other (please specify)

16. How is data entered?

- [ ] A paper questionnaire completed by a clinician
- [ ] A paper questionnaire completed by the patient
- [ ] Directly into an online web portal
- [ ] Using a tablet or mobile device in clinic
- [ ] Other (please specify)

4. Software and Data storage

17. Does your registry use an electronic data capture or storage tool (e.g. online database, or excel)

- ◯ Yes
- ◯ No

18. What software do you use as a data collection tool

- ◯ Microsoft excel
- ◯ Microsoft access
- ◯ Custom commercial solution
- ◯ Custom non-commercial solution
- ◯ Oracle
- ◯ SQL
- ◯ Other (please specify)

[ ]

19. Please describe further details of your software solution where applicable/if known

Name of software provider:	
Technical specifications	
Operation system	
Is this solution available for other users?	
Any other details	

20. Are all registrants assigned a unique identifier

- ◯ Yes
- ◯ No

21. How is the unique identifier assigned?

- ◯ Automatically
- ◯ Manually
- ◯ N/A

22. What data items are used to create the unique identifier (list all below)

5. Governance and approvals

23. Did you require approvals from an ethics board/IRB in order to set up your registry, database or mailing list?

- ○ Yes
- ○ No

24. Do participants provide informed consent before data is provided into the registry? (if possible please send a copy of the consent to elizabeth.wood2@ncl.ac.uk)

- ○ Yes- consent is implied through completion of the questionnaires
- ○ Yes- explicit consent is obtained
- ○ No

25. Is a steering committee or data access committee in place to provide governance over the registry?

- ○ Yes set up exclusively for the registry
- ○ Yes an existing group is used (e.g. Scientific advisory board or board of trustees)
- ○ No

Any comments

[ ]

26. Which of the following groups are represented on your steering or data access committee?

- ☐ Geneticists
- ☐ Clinicians
- ☐ Representative from patient organisation
- ☐ Representative from Industry/Pharmaceutical Company
- ☐ Patient or family member

6. Facilitating Research

27. Has the registry supported research in any of the following ways:

- ☐ Recruitment into therapeutic trials (Industry led)
- ☐ Recruitment into therapeutic trials (academic led)
- ☐ Recruitment into observational trials or natural history studies (Industry led)
- ☐ Recruitment into observational trials or natural history studies (academic led)
- ☐ Questionnaire mail out (post)
- ☐ Questionnaire mail out (e-mail/online)
- ☐ Provided feasibility data to commercial researchers
- ☐ Provided feasibility data to academic researchers
- ☐ Others

If yes, please provide details:

28. Please provide details of any scientific publications associated with the registry, database of mailing list below (if possible please provide PubMed reference of link to publication)

7. Registry Dataset

29. At the time of completing this survey, how many unique individuals with myotonic dystrophy are listed in your registry, database, cohort of mailing list? (Do not include information about those who have deceased if this information is collected)

Under 18 years old with myotonic dystrophy type 1 [ ]

Over 18 years old with myotonic dystrophy type 1 [ ]

Under 18 years old with myotonic dystrophy type 2 [ ]

Over 18 years old with myotonic dystrophy type 2 [ ]

30. Do you have a method to deal with duplication

- ○ yes
- ○ no

31. In 2009 a core dataset was agreed upon by experts internationally as part of a workshop sponsored by TREAT-NMD and the Marigold Foundation. Often referred to as the "Naarden dataset" . Were you aware of this dataset when setting up your registry?

- ○ Yes
- ○ No
- ○ N/A

32. Do you collect the items in the Naarden dataset?

- ○ Yes
- ○ No

* 33. Do do you collect any of the following items of the Naarden dataset? (please select all that apply)

- ☐ Sex
- ☐ First name
- ☐ Last name
- ☐ Date of Birth
- ☐ Address

- [ ] Zip/post code
- [ ] Telephone
- [ ] E-mail
- [ ] The clinical diagnosis (DM1, Congenital DM1, DM2)
- [ ] The genetic mutation result (known expansion in DMPK or CNBP)
- [ ] The size of the repeat expansion
- [ ] Method used for genetic testing (Southern blot, PCR, TP-PCR)
- [ ] Date of genetic testing
- [ ] Laboratory where test was performed
- [ ] The current best motor function (ambulant, ambulant assisted or non-ambulant)
- [ ] Wheelchair use (part-time, full-time, not at all)
- [ ] Age wheelchair use began
- [ ] Myotonia (None, mild, severe)
- [ ] Medication for myotonia
- [ ] Presence of a heart condition (arrythmia, conduction block, cardiomyopathy, other)
- [ ] Age heart condition diagnosed
- [ ] Presence of a cardiac implant (ICD or pacemaker)
- [ ] Age cardiac implant was implanted
- [ ] ECG/EKG results (PR interval, QRS duration, date of exam)
- [ ] Echocardiagram results (LVEF %, date of examination)
- [ ] Cardiac medication
- [ ] Use of invasive ventialtion
- [ ] Use of non-invasive ventilation
- [ ] Results of pulmonary function testing (FVC %, date of test)
- [ ] Presence of swallowing difficulties (dysphagia)
- [ ] Presence of gastric/nasal tube for feeding
- [ ] Cataract surgery (age at time of procedure)
- [ ] Fatigue/Excessive daytime sleepiness (none, mild, severe)
- [ ] Medication for fatigue/daytime sleepiness
- [ ] Age at onset of symptoms
- [ ] Family history of myotonic dystrophy

- [ ] Ethnic origin
- [ ] Presence in other myotonic dystrophy registry
- [ ] All of the above
- [ ] None of the above

34. From the list below, are there any items you think are **not useful and should be removed** from the Naarden dataset? (please select all that apply)

- [ ] Sex
- [ ] First name
- [ ] Last name
- [ ] Date of Birth
- [ ] Address
- [ ] Zip/post code
- [ ] Telephone
- [ ] E-mail
- [ ] The clinical diagnosis (DM1, Congenital DM1, DM2)
- [ ] The genetic mutation result (known expansion in DMPK or CNBP)
- [ ] The size of the repeat expansion
- [ ] Method used for genetic testing (Southern blot, PCR, TP-PCR)
- [ ] Date of genetic testing
- [ ] Laboratory where test was performed
- [ ] The current best motor function (ambulant, ambulant assisted or non-ambulant)
- [ ] Wheelchair use (part-time, full-time, not at all)
- [ ] Age wheelchair use began
- [ ] Myotonia (None, mild, severe)
- [ ] Medication for myotonia
- [ ] Presence of a heart condition (arrythmia, conduction block, cardiomyopathy, other)
- [ ] Age heart condition diagnosed
- [ ] Presence of a cardiac implant (ICD or pacemaker)
- [ ] Age cardiac implant was implanted
- [ ] ECG/EKG results (PR interval, QRS duration, date of exam)
- [ ] Echocardiagram results (LVEF %, date of examination)

- [ ] Cardiac medication
- [ ] Use of invasive ventialtion
- [ ] Use of non-invasive ventilation
- [ ] Results of pulmonary function testing (FVC %, date of test)
- [ ] Presence of swallowing difficulties (dysphagia)
- [ ] Presence of gastric/nasal tube for feeding
- [ ] Cataract surgery (age at time of procedure)
- [ ] Fatigue/Excessive daytime sleepiness (none, mild, severe)
- [ ] Medication for fatigue/daytime sleepiness
- [ ] Age at onset of symptoms
- [ ] Family history of myotonic dystrophy
- [ ] Ethnic orgin
- [ ] Presence in other myotonic dystrophy registry

35. Do you collect information on height and weight?

- [ ] Height
- [ ] Weight
- [ ] None

36. Do you collect additional socioeconomic data items (e.g. education, employment status)

- ( ) Yes (please specify)
- ( ) No

Please specify items collected.

[ ]

37. Do you collect any additional patient reported quality of life outcomes

- ( ) Yes (please specify below)
- ( ) No

Please specify QoL tools used

[ ]

38. Do you collect any additional patient reported pain outcomes?

- ◯ Yes (please specify below)
- ◯ No

Please specify items collected

39. Do you collect any additional patient reported fatigue outcomes?

- ◯ Yes (please specify below)
- ◯ No

Please specify items collected

40. Do you collect any additional information on gastrointestinal issues

- ◯ Yes (please specify below)
- ◯ No

Please specify items collected.

41. Do you collect any additional outcomes on the CNS involvement?

- ◯ Yes (please specify below)
- ◯ No

Please specify items collected.

42. Other than the items listed above what data is collected in the registry. (where possible please send case report forms to elizabeth.wood2@ncl.ac.uk)

* 43. To proceed please tick the box below

◯ Click here to proceed

8. Data Collection

44. What data is captured in your mailing list? (Please select all that apply)

- [ ] Sex
- [ ] First name
- [ ] Last name
- [ ] Date of Birth
- [ ] Address
- [ ] Zip/post code
- [ ] Telephone
- [ ] E-mail
- [ ] The clinical diagnosis (DM1, Congenital DM1, DM2)

45. Other than the items listed above is any other additional data captured in the mailing list. Please list here of send a copy of questionnaire/question list to elizabeth.wood2@ncl.ac.uk

9. Purpose

46. Please put the following in order of importance when thinking about the purpose of your registry, database or mailing list.

☐	Assessing disease prevalence	☐ N/A
☐	Improving standards of care	☐ N/A
☐	Recruitment into clinical research (therapeutic and observational)	☐ N/A
☐	Carrying out questionnaire based studies	☐ N/A
☐	Contacting patients about research	☐ N/A
☐	Contacting patients about care and support	☐ N/A
☐	Providing feasibility data for researchers interested in carrying out clinical research	☐ N/A
☐	Analysing disease progression (longitudinal follow up)	☐ N/A
☐	Generating hypothesis for future clinical research	☐ N/A
☐	Answering clinical questions	☐ N/A

47. Is there any additional purpose to the registry, database or mailing list, other than those listed above. Please provide any additional information of comments below.

10. Communication and Promotional materials

48. Do you use the registry, database or mailing list as a tool for communication and information dissemination with the patient community

- ○ Yes
- ○ No

49. Do you provide a newsletter to members/those registered.

- ○ Yes
- ○ No (please skip the rest of the questions on this page)

50. How do you distribute this newsletter

- ☐ Online (HTML mailing service)
- ☐ Online (Attachment to e-mail)
- ☐ Hard copy mail out
- ☐ N/A
- ☐ Other (please specify)

51. How frequently do you send out a newsletter

- ○ More than once a month
- ○ Monthly (once a month)
- ○ Quarterly (every three months)
- ○ Every six months
- ○ Annually (once a year)
- ○ Ad Hoc
- ○ N/A
- ○ Other (please specify)

52. Are other communication/promotional materials available for patients? (select all that are available)

- [ ] Leaflets for patients
- [ ] Leaflets for professionals
- [ ] Information of industry
- [ ] Other (please specify)

11. Registry experience

53. Please describe the challenges you have faced in the set up and running of your registry, database or mailing list.

54. Please describe the main benefits you have found in setting up and running your registry, database or mailing list

55. How do you think the growing number of myotonic dystrophy registries can be used to the greatest advantage of the community.

56. Would you be interested in a attending meeting to review/update registry practice?

- ○ Yes
- ○ No

Additional comments:
